# Supplementary material for: Exploring local and regional drivers of microbial biodiversity across freshwater ponds
Source: FEMS Microbiol Ecol. 2026 Jun 23;102(8):fiag068. doi: 10.1093/femsec/fiag068 (PMC13377648; doi:10.1093/femsec/fiag068)
Supplement: fiag068_Supplemental_File [file fiag068_supplemental_file.pdf]

Supplemental Material: Exploring local and regional drivers of microbial biodiversity across freshwater ponds

Authors: Emily A. Hardison[1], José F. Goyco-Blas[1], Noah T. Leith[1], Christopher J. Gabriel[1], Trina M. Wantman[2], Jakub Zegar[3], Matthew W.H. Chatfield[2], Michel E.B. Ohmer[3], Kevin D. Kohl[1*]

[1] Department of Biological Sciences, University of Pittsburgh, Pittsburgh, PA, USA
[2] School of Biology and Ecology, University of Maine, Orono, ME, USA
[3] Department of Biology, University of Mississippi, University, MS, USA

***Corresponding Author email:** kkohl@pitt.edu

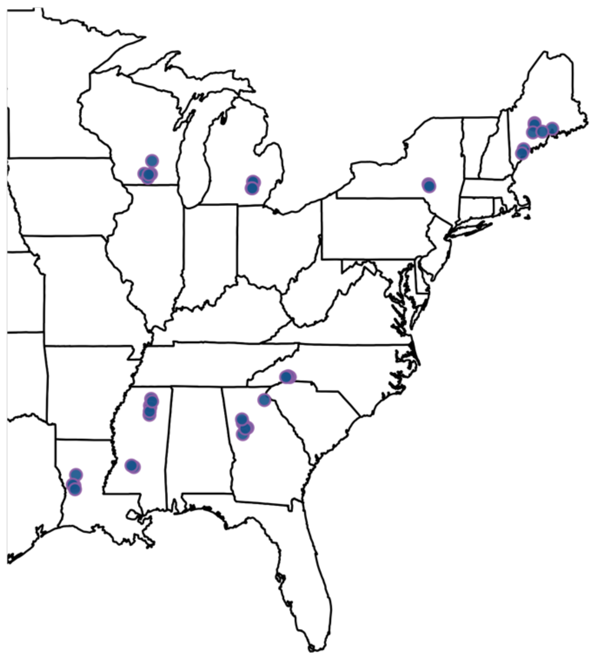

Figure S1. Site map of ponds. Please note that points were slightly jittered (width = 0.1) to improve visibility of sites near each other. Exact coordinates are provided in the associated data with the manuscript.

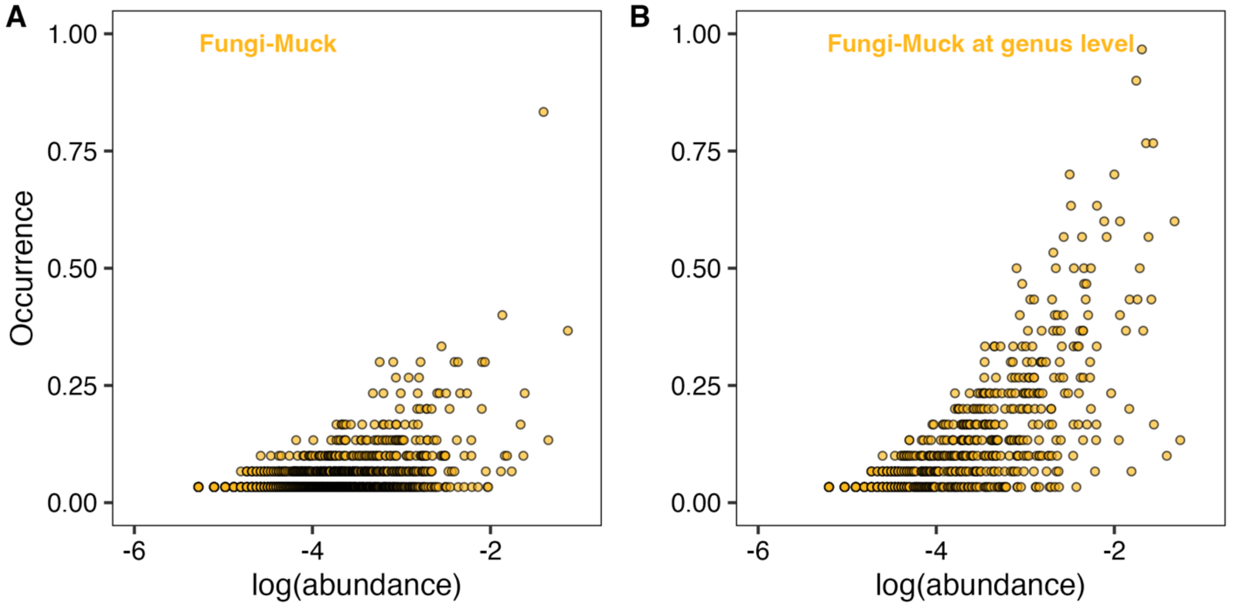

Figure S2. Comparison of abundance-occurrence patterns for pond muck fungal communities at the (A) ASV vs (B) genus level. Each point represents a different (A) ASV or (B) genus, where the occurrence (proportion of ponds containing that taxa) is plotted against the log10 of the relative abundance of the taxa.

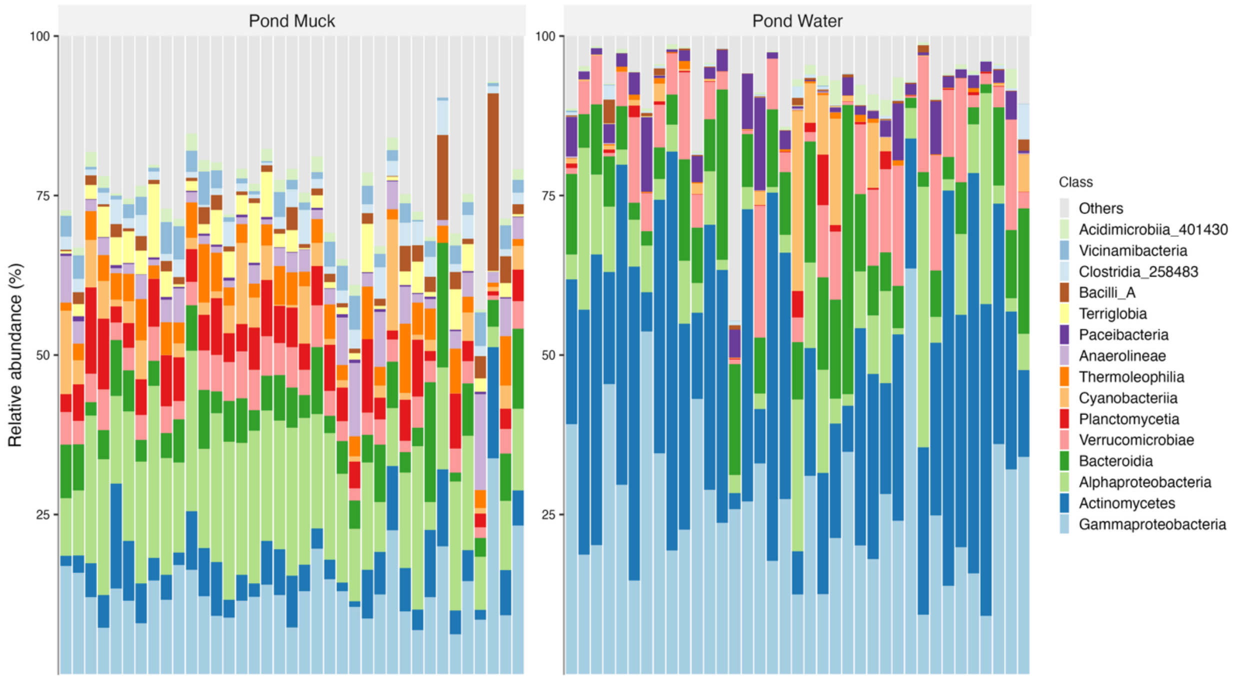

Figure S3. Taxa bar plots showing relative abundance of the top 15 most abundant classes found in pond bacterial communities. Each bar represents the community found in a different pond, with muck samples on the left panel and water samples on the right panel.

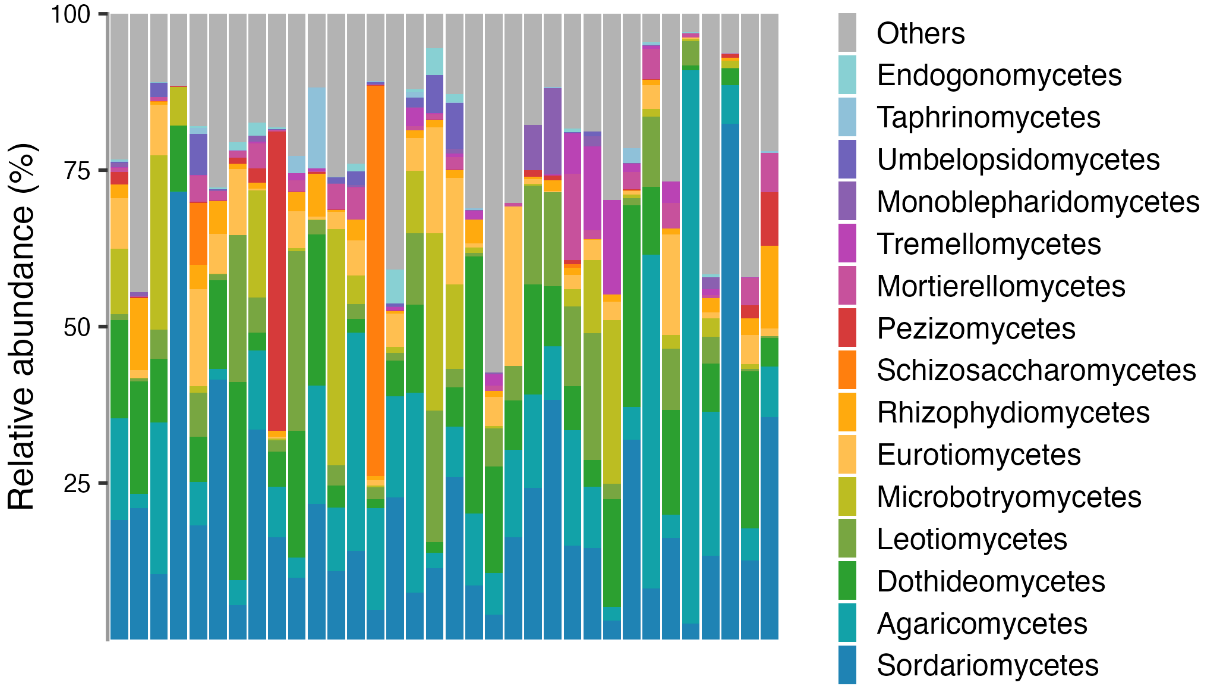

Figure S4. Taxa bar plots showing relative abundance of the top 15 most abundant classes found in pond fungal communities. Each bar represents the community found in a different pond.

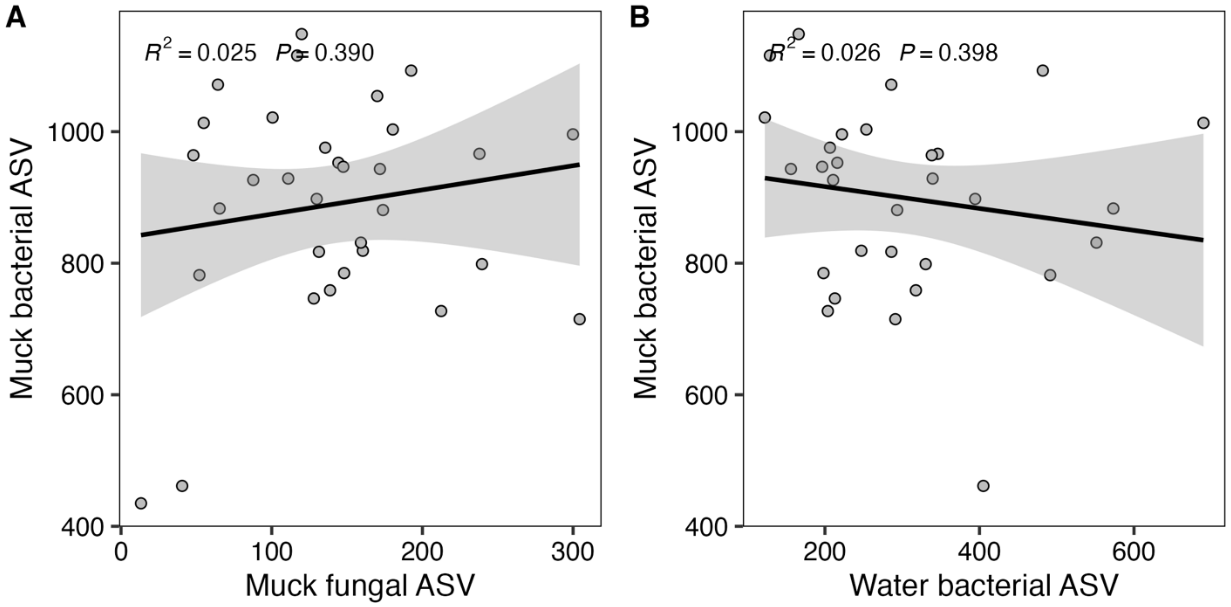

Figure S5. Comparison of ASV richness across different sample types within the same pond. **(A)** shows the relationship between muck bacterial and muck fungal richness and (B) shows muck and water bacterial richness. Lines are linear fits with confidence intervals from geom_smooth() in ggplot, with R^2 values and p-values reported on the plot.

Table S1. Significant results from ANCOM-BC2 analysis

Sample Type	Kingdom	Phylum	Class	Order	Family	Environmental variable	Log fold-change	Adjusted p-value
Muck bacterial	Bacteria	Pseudomonadota	Alphaproteobacteria	Rhizobiales_505101	Xanthobacteraceae	log10(conductivity)	-1.73	0.0416
Muck bacterial	Bacteria	Acidobacteriota	Terriglobia	Terriglobales	SbA1	log10(conductivity)	-2.53	0.008
Water bacterial	Bacteria	Pseudomonadota	Gammaproteobacteria	Methylococcales	Methylomonadaceae	Dissolved oxygen saturation (%)	-0.03	0.0088
Water bacterial	Bacteria	Cyanobacteriota	NA	NA	NA	log10(conductivity)	-3.86	0.0212
Water bacterial	Bacteria	Cyanobacteriota	Cyanobacteriia	Cyanobacteriales	Nostocaceae	Dissolved oxygen saturation (%)	0.07	0
Water bacterial	Bacteria	Cyanobacteriota	Cyanobacteriia	Cyanobacteriales	Nostocaceae	log10(conductivity)	-4.78	0.0219
Water bacterial	Bacteria	Pseudomonadota	Gammaproteobacteria	Burkholderiales	Casimicrobiaceae	Dissolved oxygen saturation (%)	0.07	8.00E-04
